# Piezoelectric Ceramics of the (1 − *x*)Bi_0.50_Na_0.50_TiO_3_–*x*Ba_0.90_Ca_0.10_TiO_3_ Lead-Free Solid Solution: Chemical Shift of the Morphotropic Phase Boundary, a Case Study for *x* = 0.06

**DOI:** 10.3390/ma10070736

**Published:** 2017-07-01

**Authors:** Rodrigo Vivar-Ocampo, Lorena Pardo, David Ávila, Emilio Morán, Amador M. González, Lauro Bucio, María-Elena Villafuerte-Castrejón

**Affiliations:** 1 Instituto de Investigaciones en Materiales, Universidad Nacional Autónoma de México, Ciudad Universitaria, A.P. 70-360, México D.F. 04510, Mexico; ocampovivar@gmail.com; 2 Instituto de Ciencia de Materiales de Madrid, CSIC, Sor Juana Inés de la Cruz, 3, Cantoblanco, 28049 Madrid, Spain; lpardo@icmm.csic.es; 3 Departamento Química Inorgánica I, Facultad de Ciencias Químicas, Universidad Complutense de Madrid, 28040 Madrid, Spain; avilad@quim.ucm.es (D.Á.); emoran@quim.ucm.es (E.M.); 4 CEMDATIC, ETSIST Campus Sur, Universidad Politécnica de Madrid, Nikola Tesla s/n. 28031 Madrid, Spain; amador@etsist.upm.es; 5 Laboratorio de Cristalofísica y Materiales Naturales, Instituto de Física, Universidad Nacional Autónoma de México, Circuito de la Investigación Científica S/N, México D.F. 04510, Mexico; bucio@fisica.unam.mx

**Keywords:** Bismuth sodium titanate, Barium titanate, solid state synthesis, Pechini synthesis route, Morphotropic Phase Boundary, lead-free, piezoelectricity, ceramics

## Abstract

Research and development of lead-free piezoelectric materials are still the hottest topics in the field of piezoelectricity. One of the most promising lead-free family of compounds to replace lead zirconate–titanate for actuators is that of Bi_0.50_Na_0.50_TiO_3_ (BNT) based solid solutions. The pseudo-binary (1 − *x*)Bi_0.50_Na_0.50_TiO_3_–*x*Ba_1 − *y*_Ca_y_TiO_3_ system has been proposed for high temperature capacitors and not yet fully explored as piezoelectric material. In this work, the solid solution with *x* = 0.06 and *y* = 0.10 was obtained by two different synthesis routes: solid state and Pechini, aiming at using reduced temperatures, both in synthesis (<800 °C) and sintering (<1150 °C), while maintaining appropriated piezoelectric performance. Crystal structure, ceramic grain size, and morphology depend on the synthesis route and were analyzed by X-ray diffraction, together with scanning and transmission electron microscopy. The effects of processing and ceramic microstructure on the structural, dielectric, ferroelectric, and piezoelectric properties were discussed in terms of a shift of the Morphotropic Phase Boundary, chemically induced by the synthesis route.

## 1. Introduction

Solid solutions based on PbTiO_3_, among others, the one containing lead zirconate (PbZrO_3_), also known as PZT, dominate industrial applications of ferroelectrics in the polycrystal form. PZT production involves the introduction of toxic lead oxide into the environment. European Union regulations arising in 2003 triggered the study of lead-free piezoelectric compositions [1]. This research field has been growing rapidly ever since [2]. Today it has reached a level of maturity in some scientific themes and industrial transference [3]. Compositions within the Morphotropic Phase Boundary (MPB) [4], like the one of PZT, are the most sought after because of the coexistence of the ferroelectric polymorph crystal structures, which are stable at each side of the boundary, The reason is that the number of favorable directions of the spontaneous polarization increases for the MPB and ceramics can achieve the best properties [5] by application of an external electric field (poling).

Among the lead free ceramic families, bismuth sodium titanate, Bi_0.5_Na_0.5_TiO_3_ (BTN)-based solid solutions, especially BNT-rich side compositions, have been the focus of interest due to the fact that they possess MPBs and to their promising properties [6,7]. BNT ceramics have been studied since the early 60s [8]. BNT is a ferroelectric relaxor, with a large remnant polarization, Pr = 38 μC/cm^2^, a large coercive field, Ec = 73 kV/cm, and a high Curie temperature, *T*_C_ = 320 °C. The disordered A-site substituted perovskite-type structure of BNT has a sequence of phase transitions on cooling from high temperature paraelectric-cubic (C) to antiferroelectric-tetragonal-Pb4m (AFE-T) to room-temperature ferroelectric-rhombohedral-R3c (R) symmetries [9]. The T and R phases coexist over a rather broad interval of temperatures [9]. In addition to this structural complexity, non-stoichiometry affects both structure [10] and properties [11]. At sintering temperatures (*T*_s_ > 1000 °C) Bi_2_O_3_ and Na_2_O are volatiles, which result in changes in the stoichiometry. The volatility issue for BNT processing was approached using lower temperatures and shorter heating times for synthesis and sintering [12,13]. Besides, inducing the piezoelectric effect in the randomly oriented ceramic by orientation of ferroelectric domains under an external electric field (poling) is difficult due to its large coercive field and relatively large conductivity, thus limiting its use as a piezoceramic. In order to improve their properties and avoid some of these disadvantages, numerous binary and ternary solid solution systems with MPB compositions have been proposed [4].

In particular, the solid solution (1 − *x*)Bi_0.50_Na_0.50_TiO_3_–*x*BaTiO_3_, studied for the first time in the early 90s [14], has been of special interest because of its MPB that separates poled ferroelectrics of BNT-rich compositions with rombohedral-R3c structure (*x* < 0.06) from BT-like ferroelectric-tetragonal-P4mm (FE-T) structured ones (*x* > 0.06). After years of research, these ceramics have proved superior to PZTs as actuators due to the giant strain [15], up to 0.7% with low hysteresis [16], with respect to the typical 0.2% of remnant strain in PZT [15]. Such a high value is a consequence of an electric field-induced phase transition from the relaxor state of the unpoled ceramics to the coexistence of ferroelectrics at the MPB after poling [17].

**Figure 1:**
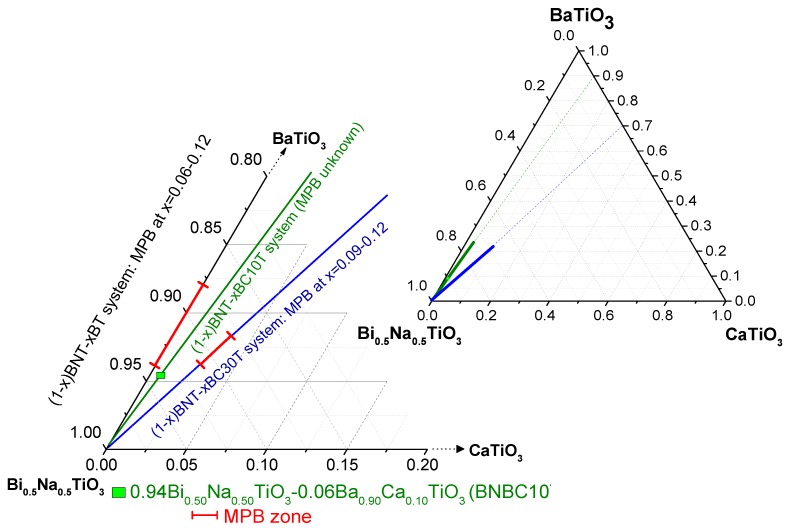
Ternary diagram of the (1 − *x*)Bi_0.50_Na_0.50_TiO_3_–*x*Ba_1 − *y*_Ca*_y_*TiO_3_ system, summarizing the results in literature and the composition studied in this work. Morphotropic Phase Boundary (MPB)

On the one hand, the synthesis of BT usually requires relatively high temperatures (*T* > 1300 °C), therefore, synthesis issues became a key factor to produce (1 − *x*)BNT–*x*BT (BNBT100*x*) ceramics with desired performance [18]. On the other hand, both BT and their isovalent substitutions, among them Ba_1 − *y*_Ca_y_TiO_3_ (BCT), are also considered candidates for ceramic capacitor material, including multilayer ceramic capacitor, in which the temperature stability and reliability of the dielectric properties of the pure BT have been improved [19,20,21,22]. Consequently, it is meaningful to study compositions at the pseudo-binary system (1 − *x*)Bi_0.50_Na_0.50_TiO_3_–*x*Ba_1 − *y*_Ca_y_TiO_3_ (or BNBC100yT100*x*) (Figure 1) as lead-free piezoceramic in order to achieve processing at reduced temperature. Though processing of some of these compositions has been achieved and proposed for high temperature capacitors [23], this solid solution has not yet been fully explored as a piezoelectric material.

Noteworthy, (1 − *x*)Bi_0.50_Na_0.50_TiO_3_–*x*(Ba_0.70_Ca_0.30_)TiO_3_ (with *x* = 0, 0.03, 0.06, 0.09, 0.12, 0.15) [24] was obtained by conventional solid state synthesis and sintered at 1100–1190 °C for 2 h in covered alumina crucibles. Authors claim that X-ray diffraction (XRD) of unpoled ceramics reveals a MPB between a ferroelectric rhombohedral phase for x < 0.09 and a ferroelectric tetragonal phase for x > 0.12. Notwithstanding the assumption of a MPB in that work, a moderate value of d_33_ = 125 pC/N for x = 0.09 was reported. This value must be compared with those obtained for other systems with coexistence of two ferroelectric phases at MPB, e.g., d_33_ = 220 and 593 pC/N for pure and modified commercial lead titanate zirconate PZT48/52 and PZT5H, respectively [5]; d_33_ = 160 and 570 pC/N for pure [5] and modified KNN [25] respectively; d_33_ = 620 pC/N for Ba(Zr_0.15_Ti_0.85_)O_3_–*x*(Ba_0.8_Ca_0.2_)TiO_3_ [26].

The purpose of this work is to process ceramics of 0.94Bi_0.50_Na_0.50_TiO_3_–0.06Ba_0.90_Ca_0.10_TiO_3_ composition (hereafter referred to as BNBC10T6) and their study as reliable materials for piezoelectric applications. We aimed to use soft synthesis and sintering conditions and compare the performance of ceramics prepared by mixed oxides and the Pechini method as an alternative route. Structural studies were carried out comparatively with recently published results on BNBT6 [27] to assess the coexistence of polar and weakly-polar phases that characterize BNT-based compositions at the rhombohedral edge of the MPB. Dielectric and piezoelectric properties were studied and discussed in terms of a shift of the MPB, chemically induced by the synthesis route.

## 2. Results and Discussion

### 2.1. Structural and Electrical Characterization of Ceramics from the Solid State Route

**Figure 2:**
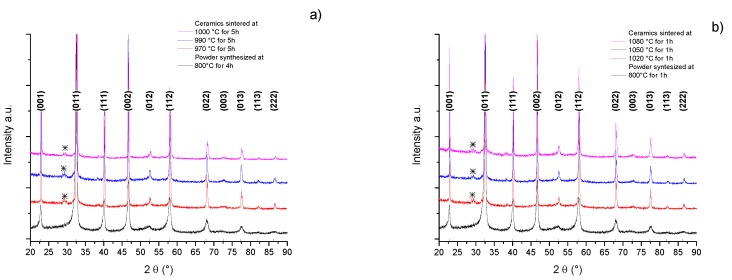
Solid state synthesis. X-ray diffraction (XRD) patterns (before k_α2_ correction) of powder and ceramic samples for: (**a**) low temperature sintering after synthesis at 800 °C—4 h and (**b**) high temperature sintering after synthesis at 800 °C—1 h. * Secondary phases.

The X-ray diffraction (XRD) patterns of BNBC10T6 calcined powders obtained by solid state reaction at 800 °C for 1 h and 4 h and those of the corresponding sintered ceramics at different temperatures are shown in Figure 2. Powder samples and ceramics showed a perovskite-type crystal structure [9]. Powder presents low intensity wide peaks and ceramics show high and sharp peaks indicating grain growth during sintering. All ceramics present traces of secondary phases near 2θ = 30°, marked with asterisk. The diffraction peaks of the samples were indexed to the cubic perovskite prototype structure. Sintering at higher temperatures but with conventional lower times does not reduce the second phase content.

**Figure 3:**
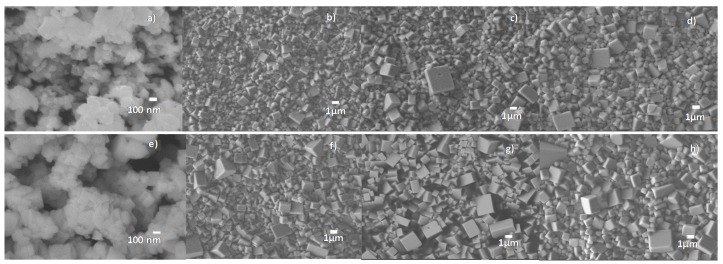
Scanning electron microscopy (SEM) photographs of BNBC10T6 samples obtained by solid state method. Powder synthesized at (**a**) 800 °C for 4 h and corresponding ceramics prepared by low temperature sintering at (**b**) 970 °C; (**c**) 990 °C and (**d**) 1000 °C for 5 h. Powder synthesized at (**e**) 800 °C for 1 h and corresponding ceramics prepared by high temperature sintering at (**f**) 1020 °C; (**g**) 1050 °C and (**h**) 1080 °C for 1 h.

The BNBC10T6 ceramic microstructure of solid state powder and ceramics was analyzed by scanning electron microscopy (SEM) and the results are given in Figure 3. After synthesis at 800 °C—4 h, the low temperature sintering series of ceramics achieved just slightly lower grain size than those prepared at higher sintering temperatures and conventional lower times. For all samples, there is a characteristic cubic morphology of the ceramic grains and a low porosity (94–96% densification). The highest homogeneity in grain size is observed for the sample sintered at 970 °C—5 h after synthesis at 800 °C for 4 h (Figure 3b).

Table 1 summarizes the preparation conditions, density, as well as the piezoelectric and dielectric parameters of the poled ceramics obtained by the solid state route. For both series of ceramics, the lower electromechanical activity measured at resonance (d_31_, k_p_) corresponds to the samples that show higher dielectric losses. The ceramics of the high sintering series show higher relative permittivity. The highest d_33_ is measured for the sample sintered at 970 °C for 5 h. The piezoelectric parameters are not as high as expected for a MPB composition with coexistence of ferroelectric phases.

### 2.2. Structural and Electrical Characterization of Ceramics from the Pechini Route

**Figure 4:**
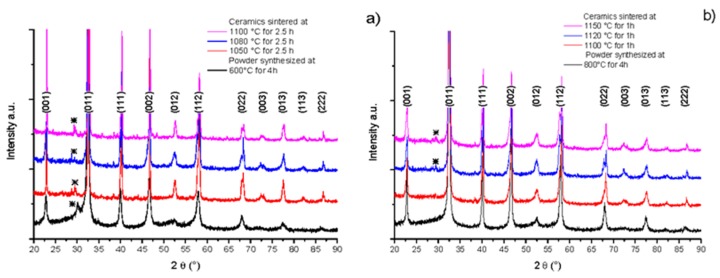
Pechini synthesis method, XRD pattern (before k_α2_ correction) of powder and ceramic samples for: (**a**) low temperature sintering after synthesis at 600 °C—4 h and (**b**) high temperature sintering after synthesis at 800 °C—4 h. * Secondary phases.

XRD patterns of BNBC10T6 powder synthesized by the Pechini method and the corresponding sintered ceramics are shown in Figure 4. Powder samples and ceramics showed a perovskite-type crystal structure, indexed to the cubic prototype structure [9]. Powder presents low intensity wide peaks, while ceramics show high and sharp peaks revealing the grain growth after sintering. Figure 4a shows that both the powder with heat treatment at 600 °C—4 h and the corresponding ceramics present traces of secondary phases that cannot be eliminated by sintering at 1100 °C—2.5 h.

Figure 4b indicates that the powder treated at 800 °C—4 h and the ceramic sintered at 1100 °C—1 h prepared from it do not present traces of secondary phases, but the ceramics sintered at higher temperatures does (Figure 4b). This is a sign of a degradation or decomposition of the material due to excessively high temperature treatment.

**Figure 5:**
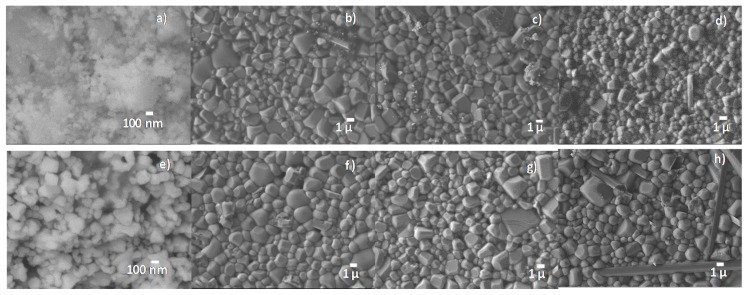
SEM photographs of BNBC10T6 samples obtained by the Pechini method. Powder synthesized at (**a**) 600 °C for 4 h and corresponding ceramics sintered at: (**b**) 1050 °C; (**c**) 1080 °C and (**d**) 1100 °C for 2.5 h. Powder synthesized at (**e**) 800 °C for 4h and corresponding ceramics sintered at: (**f**) 1100 °C; (**g**) 1120 °C and (**h**) 1150 °C for 1 h.

The BNBC10T6 grain size and morphology of the Pechini synthesis powder and the ceramics prepared from it were analyzed by SEM (Figure 5). In contrast to the solid state ceramics, the grain morphology of these ceramics tends to be spherical giving place to a good packing of grains and low porosity of the ceramic bodies (94–96% densification). All these ceramics present more homogeneous microstructures than those of solid state, lacking the big grains (size > 3 µm) observed in most of the solid state samples (Figure 3). Again, ceramics sintered at low temperature achieve similar grain size to those prepared with higher temperatures and conventional lower times.

Table 2 summarizes the preparation conditions, density, as well as the piezoelectric and dielectric parameters of the poled ceramics obtained by the Pechini route. These ceramics present lower dielectric permittivity and losses than those from the solid state route, though the mean grain size seems to be systematically lower than for solid state ceramics. This is most probably a consequence of the microstructures without big grains (Figure 3 and Figure 5) for Pechini ceramics, but may also be revealing a crystal structure or compositional difference between the two types of ceramics. Though the d_33_ coefficients are lower than for the ceramics from solid state, the electromechanical parameters at resonance (d_31_, k_p_) are higher; lower d_33_ and higher electromechanical coupling factors than for the MPB [7,28]. As it was observed for the ceramics from the solid state synthesis, the piezoelectric parameters of the ceramics from the Pechini route are not as high as expected for an MPB with coexistence of ferroelectric phases.

### 2.3. Comparative Structural Analysis of Ceramics from the Solid State and Pechini Routes: XRD

**Figure 6:**
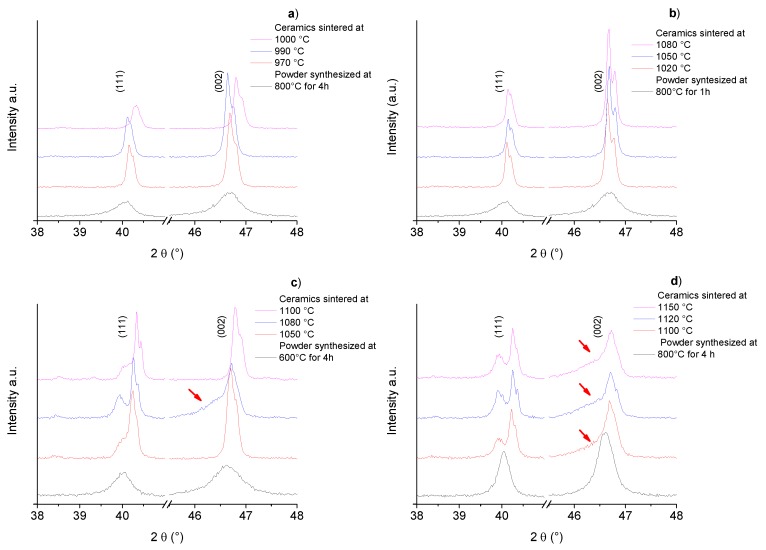
Structural features of the XRD 111 and 002 perovskite peaks (before k_α2_ correction) of ceramics prepared from synthesis by solid state (**a**,**b**) and Pechini (**c**,**d**).

Many works claim that there is coexistence of ferroelectric-rhombohedral-R3c and ferroelectric-tetragonal-P4mm symmetries at the rhombohedral edge of the MPB of the BNT-BT and similar solid solution systems. This claim is often based on the splitting of the perovskite cubic prototype peaks, e.g., the 111 and 002 peaks. In Figure 6 we can see selected angular ranges of the XRD patterns for all the studied ceramics showing such splitting. Nevertheless, the corresponding increase in piezoelectric properties of ceramics in the solid solution systems of (1 − *x*)(Bi_0.50_Na_0.50_)TiO_3_–*x*BaTiO_3_ (BNBT100*x*) [14] and (1 − *x*)(Bi_0.50_Na_0.50_)TiO_3_–*x*(Ba_0.70_Na_0.30_)TiO_3_ (BNBC30T100*x*) [24] has not been documented.

As an alternative model to explain the splitting of the XRD peaks before the application of an electric field, a multisymmetric crystal structure has been proposed for BNBT6 ceramics prepared by sol-gel autocombustion synthesis. This model was obtained from the analysis of high resolution X-ray diffraction patterns of synchrotron radiation [27]. It consists of: (a) a global ferroelectric-rhombohedral R3c symmetry; (b) a globally non-polar cubic-Pm-3m symmetry, which presents intense but wide peaks, and (c) a minor nanosized phase that takes into account the observed wide peaks at the low-angle side of some of the perovskite peaks (marked with arrows in Figure 6) for the Rietveld model.

It must be clarified that XRD alone does not allow the local symmetry of the globally non-polar cubic Pm-3m symmetry of this model to be determined nor the one of the minor nanosized phase, which is mainly characterized by relatively weak peaks. For this, complementary studies, e.g., by Transmission Electron Microscopy (TEM) [29] or high resolution neutron diffraction [30] techniques must be carried out. Nanosized regions of FE-R3c and AFE-P4bm and, therefore, the existence of local polar order, have been observed for BNBT6 [30]. Centro-symmetry at local level for the globally non-polar cubic phase was also discharged by X-ray Absorption Near Edge Spectroscopy (XANES) analysis for BNBT6 ceramics prepared by sol-gel autocombustion [27].

The fraction of the ferroelectric R3c symmetry in BNBT6 ceramics increases after application of an electric field at the expense of the Pm-3m cubic symmetry [31]. That is, the short-range polar order (nanodomains) of the globally non-polar component of the material becomes a long-range polar order (macroscopic ferroelectric domains) under the action of the electric field. As a consequence of the change in the polar structure, the dielectric permittivity also changes [17].

It must be also clarified that this electric-field-induced phase transition is the main characteristic that defines the rhombohedral edge of the MPBs in these BNT-based solid solutions and that makes them different from the narrow MPB with coexistence of two ferroelectric phases before poling, like the one of PZT. However, the coexistence of ferroelectric rhombohedral R3c and ferroelectric tetragonal P4mm is well documented at the tetragonal edge of the 0.08 < x < 0.11 [15]. Besides, the wide peaks at the low-angle side of the strong perovskite peaks (marked with arrows in Figure 6) disappear after powdering the poled samples, thus indicating that this is a structural feature related to a surface structure [31] but not to the coexistence of ferroelectric phases.

In Figure 6 we can see that the three mentioned features observed at XRD patterns of BNBT6 sol-gel autocombustion ceramics [27,31], are not observed in all samples. Specifically, the wide peaks associated with a surface structure [27,31,32,33] are only observed in most of the ceramics prepared by the Pechini route (marked with arrows). Therefore this feature seems to be dependent on the synthesis route and sintering conditions as well as on the resulting grain size and morphology, porosity (Figure 3 and Figure 5) and stoichiometry of the ceramics.

**Figure 7:**
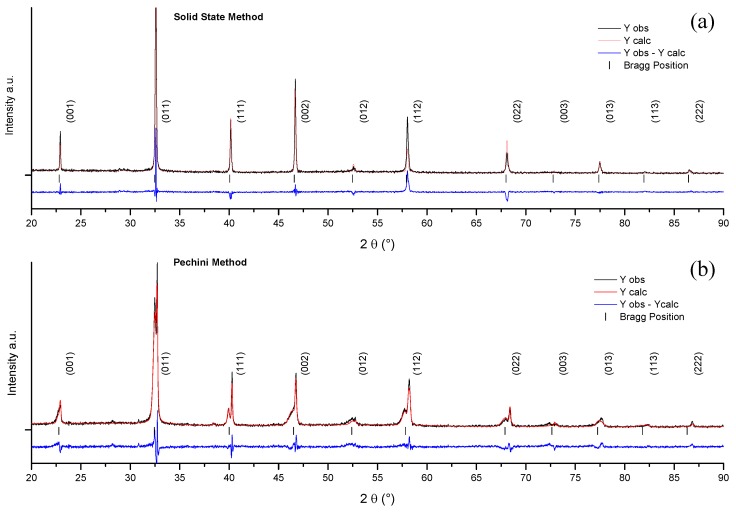
Measured and Rietveld calculated XRD patterns of as-sintered ceramics from solid state synthesis at 800 °C—4 h with sintering at 970 °C—5 h (**a**) and from Pechini synthesis at 600 °C—4 h with sintering at 1080 °C—2.5 h (**b**).

**Figure 8:**
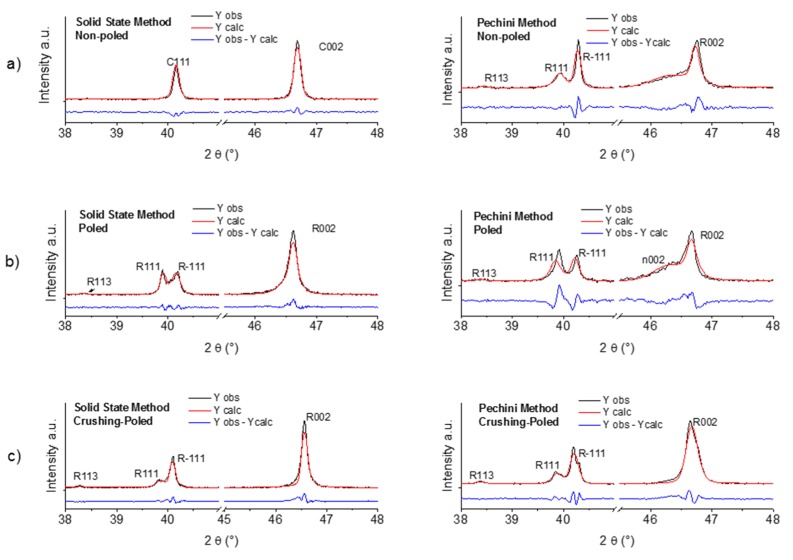
Measured and Rietveld calculated 111 and 002 perovskite peaks. In comparison, the analysis of ceramics from solid state synthesis at 800 °C—4 h with sintering at 970 °C—5 h and from the Pechini route at 600 °C—4 h with sintering at 1080 °C—2.5 h. For both types of materials, the studied samples are: (**a**) as-sintered, non-poled; (**b**) poled; and (**c**) poled and, afterwards, powdered samples.

To determine the structural model of BNBC10T6 ceramics studied here on the basis of the recently published results on BNBT100*x* [27,31,34], we also investigated the low temperature sintered samples having the highest d_33_ coefficient from the solid state and from the Pechini synthesis routes, sintered at 970 °C—5 h (Table 1) and at 1080 °C—2.5 h (Table 2), respectively. Rietveld analysis of XRD patterns was carried out after corrections by k_α2_ [35]. Flat sintered ceramic specimens, before and after poling, and powder obtained by crushing the poled ceramics were studied. Results of this analysis are shown in Figure 7 and Figure 8 and in Table 3 which exhibits the lattice parameters and fractions of the symmetries involved in the model, as well as the quality factors of the Rietveld refinements.

The as-sintered ceramic from solid state, after pattern correction by k*_α_*_2_, presents a cubic perovskite structure. It shows intense well-defined peaks without any splitting (Figure 7a and Figure 8a). This cubic structure was also observed by many authors in BNBT6 ceramics before poling [36,37]. It was modeled in this work as a single Pm-3m [36], globally non-polar symmetry. It must be noted that this composition, according to the literature, should be at the rhombohedral, BNT-structure type, left side of the MPB, but close to the edge of the wide MPB, as it is observed for BNBT6 [27,29,30,37] (Figure 1).

After poling, there is a major change in the crystal structure of the ceramic from the solid state synthesis. The clear splitting of the 111 cubic peak into the R111 and R-111 peaks (Figure 8b) and the small R113 peak at the low-angle side of the 111 doublet, shows that the ceramic is transformed into a field-induced ferroelectric rhomboedral-R3c structure. Table 3 presents the crystal lattice distortion of this field-induced ferroelectric, a = 3.9006 Å and α = 89.76°, which has a preferential orientation in the direction of the polar axes, revealed by a peak intensity ratio I_111_/I_−__111_ ≈ 1 (Figure 8b). This is a strong cell distortion of this field-induced ferroelectric in comparation with the values for pure BNT (a = 3.89 Å, α = 89.84°) and BNBT6 (a = 3.90 Å, α = 89.70°, space group R3c) [31].

After the poled sample is powdered such preferential orientation is lost, due to the random orientation of powder particles of the poled ceramic (Figure 8c).

In contrast to the results of the solid state ceramic, the as-sintered ceramic from the Pechini route, after pattern correction by k_α2_, has a more complex structure (Figure 7b and Figure 8a). Before poling, the ceramic from Pechini route shows a ferroelectric rhombohedral-R3c structure, revealed by the splitting of the 111 cubic peak and the R113 peak. This XRD pattern (Figure 7b) was modeled as a R3c symmetry together with a fraction of a nanosized phase, with cubic Pm-3m symmetry, to take into account the observed wide peaks at the low-angle size of the perovskite peaks (marked as n002 in Figure 8a). This is the same structure as the one found for BNBT4 ceramics from sol-gel autocombustion [34]. Table 3 shows the parameters of the global rhombohedral phase, a = 3.8928 Å and α = 89.63°, which again indicate the strong distortion of the cell of this spontaneous ferroelectric in comparation with the values for pure BNT.

After poling (Figure 8b), the main changes in the structure of the ceramic from the Pechini synthesis are: (a) the increase in rhombohedral distortion (α_poled_ = 89.61°) and (b) the preferential orientation of the ceramic in the direction of the polar axis. The orientation is shown by the increase in the peak intensity ratio I_111_/I_−__111_ with respect to the as-sintered sample (Figure 8a,b), and it is due to ferroelectric domain orientation. After poling, there is also a small increase (<1%) in R3c symmetry at the expense of Pm-3m phase. After the poled sample is powdered, the nanosized phase is strongly reduced (from 50% to 15%) and the ferroelectric R3c symmetry becomes majority; there is a decrease in this distortion and its preferential orientation is lost.

Overall, this analysis reveals that, while BNTBC10T6 ceramics obtained by the Pechini synthesis route are at the rhombohedral side of the MPB, BNTBC10T6 ceramics from the solid state synthesis with a structure of the rhombohedral edge of the MPB are obtained, because they present a field-induced phase transition with change in the crystal symmetry. Therefore, the structural characteristics of the BNTBC10T6 ceramics seem to be dependent on the synthesis route. This finding is for the first time reported here for bulk ceramics at the BNT-based systems, characterized by a high structural complexity. However, other examples of dependence of the crystal structure on the processing and microstructure are found in the literature for other ferroelectric solid solutions [38,39,40].

### 2.4. Comparative Structural Analysis of Ceramics from the Solid State and Pechini Routes: TEM

**Figure 9:**
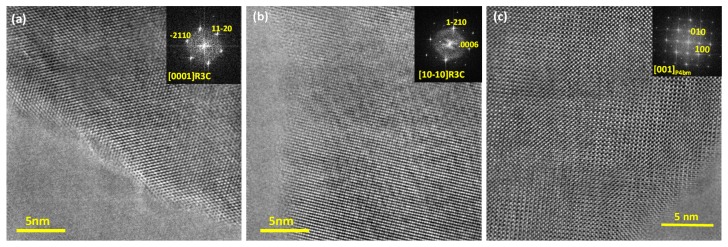
High resolution Transmission Electron Microscopy (TEM) images for the ceramic obtained by solid state route along the (**a**) [0001] and (**b**) [10−10] zone axis of crystals with R3c symmetry and along (**c**) [001] for the crystals showing P4bm symmetry. The indexed digital diffraction patterns of each image is displayed in the top right as inset.

**Figure 10:**
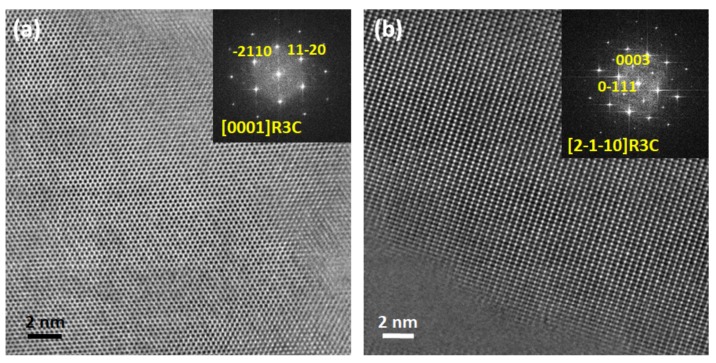
High-resolution TEM images and their corresponding digital diffraction patterns for the ceramic obtained by the Pechini route, well crystallized and a major with R3c symmetry along the main (**a**) [0001] and (**b**) [2−1−10] zone axis.

Powdered samples before application of the electric field were analyzed by TEM. High-resolution micrographs and their corresponding digital diffraction patterns are shown in Figure 9 and Figure 10 for the ceramics from the solid state and Pechini routes, respectively.

The solid state ceramic, globally cubic as for XRD (Figure 7a), presents two types of crystallites. Majority zones with FE-rhombohedral R3c symmetry were found, but also a minor proportion with AFE-tetragonal P4bm symmetry. Similarly, coexistence of FE-R3c and AFE-P4bm zones with nanometric size was reported for BNBT6 ceramics structure at the rhombohedral edge of the MPB before the application of an electric field, after which a field-induced FE-rhombohedral structure was observed [30].

The Pechini ceramics present only zones with rhombohedral R3c symmetry, which confirms that the wide peaks (marked with arrows in Figure 6) do not contribute to the bulk structure of the material. This result also confirms that the Pechini ceramics have a crystal structure that corresponds to the rhombohedral, BNT-rich side, of the MPB.

### 2.5. Comparative Dielectric Permittivity Curves of Ceramics from the Solid State and Pechini Routes

Phase transitions that involve a change of the polar state (from non-polar to polar or from a short-range polar order to a long-range one) of the crystals are accompanied by a subsequent change of dielectric permittivity. The measurement of dielectric permittivity curves as a function of temperature at different frequencies on heating above the transition to the para-electric state and on cooling provides information about the local structure at room temperature [7,17,41] and about the reversibility and hysteresis of phase transitions.

**Figure 11:**
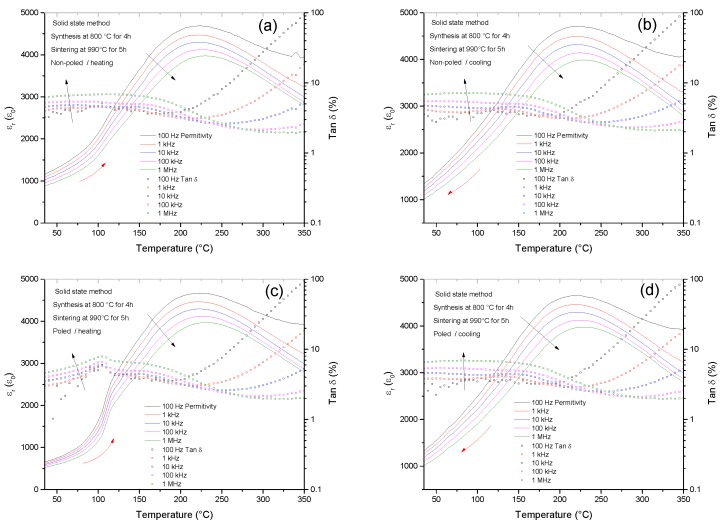
Ceramic from the solid state route. Dielectric permittivity and losses as a function of the temperature on heating for the (**a**) non-poled and (**c**) poled ceramics. (**b**,**d**) are measurements on cooling for the non-poled and poled ceramics, respectively. Red arrows indicate heating and cooling and black arrows indicate increasing frequency of the measurement.

Dielectric permittivity and losses as a function of the temperature on heating and on cooling for the non-poled and poled ceramics obtained by the solid state route are shown in Figure 11. The measurement of the non-poled sample on heating does not show a sharp change of slope of permittivity or local maxima of the dielectric losses. It shows a frequency dependence from room temperature to the maximum at ~220 °C. This is a typical relaxor behavior related to the presence of polar nanoregions [42], which indicates a short-range polar order. Poling induces a ferroelectric phase, a long-range polar order (Figure 8b) with lower frequency dependence, which has a transition to the relaxor phase at ~100 °C. This transition is observed in the peak of the dielectric losses and abrupt increase in permittivity. The result of the poled sample on cooling, which is identical to the non-poled one, indicates that the transition at ~100 °C is irreversible. This dielectric behavior is repeated for all the solid state samples studied and it is identical to the one that has been repeatedly described in the literature for BNBT6 [17,41] at the rhombohedral edge of the MPB.

**Figure 12:**
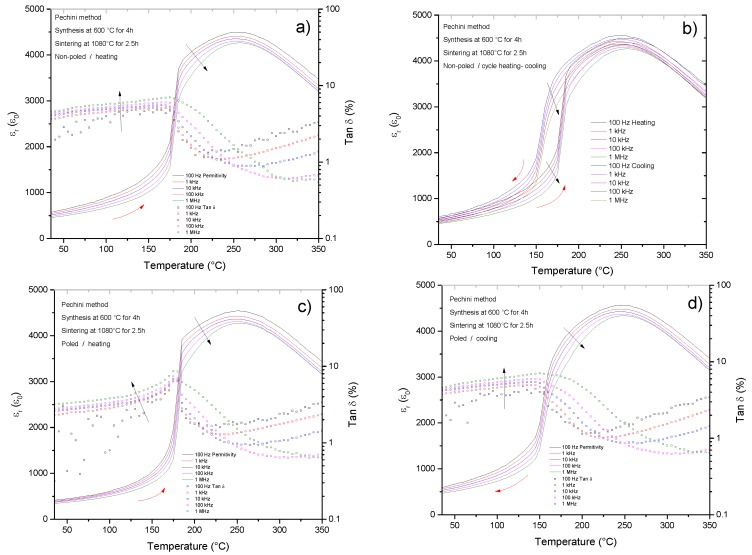
Ceramic from the Pechini route. Dielectric permittivity and losses as a function of the temperature on heating for the (**a**) non-poled and (**c**) poled ceramics. Measurements on cooling for the (**b**) non-poled and (**d**) poled ceramics. Red arrows indicate heating and cooling and black arrows indicate increasing frequency of the measurement. (**b**) Shows also the thermal hysteresis of the phase transition from (on-heating) and to (on-cooling) the spontaneous room temperature ferroelectric is shown in (**b**).

Figure 12 shows the permittivity curves on heating and cooling for the non-poled and poled samples obtained by the Pechini route that show a main ferroelectric rhombohedral spontaneous structure. The measurement for the spontaneous ferroelectric non-poled sample on heating shows a very sharp increase in permittivity and local maxima of the dielectric losses at ~175 °C. These two features of the curve are the same for the poled ceramic; they just become sharper. The transition from the high temperature phase to the room temperature spontaneous ferroelectric is reversible and shows a hysteretic nature; it takes place on cooling at ~150 °C. All the curves show a slight degree of frequency dependence which indicates, even in the ferroelectric poled samples that there is a fraction of polar nanoregions (PNRs) [42]. This dielectric behavior is repeated for all the Pechini samples studied and it is identical to those widely reported in literature for BNBT4 [7,41] at the rhombohedral side of the MPB.

Overall, this dielectric behavior is in agreement with the previous structural and electromechanical measurements and confirms the conclusions obtained from them.

As shown before, BNBC10T6 dense ceramics were prepared by two different chemical routes, leading to different microstructure (Figure 3 and Figure 4), different crystal structure (Figure 7, Figure 9, and Figure 10) and different dielectric permittivity dependence of the temperature (Figure 11 and Figure 12). If these results could be explained on the basis of compositional differences between the two types of ceramics, BNBT6-like properties would be observed for the Pechini-route ceramics. These are prepared at lower temperature and, consequently, would experience lower losses of volatiles which are the main reason for deviation from the nominal composition. Our results contradict this explanation. However, the chemical shift of the MPB can be explained, in agreement with the publications previously mentioned [39,40] and the well-known fact that, for a given composition, the equilibrium polymorph for the material structure and the related properties depend not only on the external electric field and temperature, but also on the mechanical stress.

In comparison with the literature (Figure 1), it was expected that the BNBC10T6 composition were at the rhombohedral side of the MPB, but close to the edge of the wide MPB, as is observed for BNBT6 [17,27,29,30,37].

If BNBC10T6 were at the edge of the MPB, it is expected that its crystalline structure would be globally cubic (Figure 7 and Table 3) and locally polar, consisting of regions with ferroelectric rhombohedral (FE-R3c) and antiferroelectric-tetragonal (AFE-P4bm) symmetry (Figure 9). It is also expected to have a dielectric relaxor behavior up to the transition temperature to the paraelectric phase (Figure 11). From such a globally cubic structure, it is expected that a global rhombohedral (R3c) ferroelectric (Figure 8 and Table 3) would be aroused after application of an electric field (Figure 8 and Table 3), consequently, showing piezoelectric properties (Table 1). It is also expected that upon heating the field-induced BNBC10T6 ferroelectric would have a phase transition at 100 °C to a relaxor phase (Figure 11). All these expected characteristics were indeed found for BNBC10T6 ceramics prepared by the solid state route.

However, the properties of BNBC10T6 ceramics prepared by the Pechini route differ from the above described expected behavior. The properties of ceramics from the Pechini route correspond to those found for spontaneous ferroelectric (Figure 12) with globally rhombohedral (R3c) compositions (Figure 7 and Table 3), as is observed, e.g., for BNBT4 [7,29,37,41].

The different precursors used in the two synthetic routes here considered give place to the BNBC10T6 perovskite through different reaction routes and byproducts that affect the mass transport during sintering and determine the ceramic microstructure and, from this, the final properties. It has been observed previously that fine-grain ceramic microstructures stabilize a ferroelectric structure in the antiferroelectric NaNbO_3_ [39,40] The mechanisms underlying this structural stabilization involve the existence of intragranular stresses. These stresses are induced by the decreased compensation of the ferroelastic energy during the formation of non-180° domain walls as the grain size decreases. Similarly, and due to the compact ceramic microstructure with lower grain size observed for Pechini ceramics (Figure 5), together with the relatively low distortion from the cubic prototype that facilitates the change of polymorph, the mixed local structure (FE-R3c and AFE-P4bm) of the solid state BNBC10T6 ceramics (Figure 9) turns into uniform FE-R3c phase of the Pechini BNBC10T6 ceramics by stabilization of the ferroelectric structure in the AFE-P4bm zones.

All the experimental results here reported mean that the expected behavior for the studied composition, which was observed for the BNBC10T6 ceramics of the solid state route here studied, will be observed for the BNBC10T100*x* ceramics of the Pechini route for x > 0.06. Therefore, our results reveal a, microstructure mediated, chemical shift of the MPB in the BNBC10T100*x* solid solution.

## 3. Experimental Method

### 3.1. Materials Preparation

Powder of nominal 0.94(Bi_0.50_Na_0.50_TiO_3_)–0.06Ba_0.90_Ca_0.10_TiO_3_ composition (hereafter referred to as BNBC10T6) was produced by two different routes: the solid state method and Pechini route. In the solid state method, the reagent grade raw materials Bi_2_O_3_ (Sigma-Aldrich, St. Louis, MO, USA, 99.9%), Na_2_CO_3_ (Fermont, Monterrey, México, 99.9%), TiO_2_ (Sigma-Aldrich 99%), BaCO_3_ (Sigma-Aldrich 99%), and CaCO_3_ (Fluka, Seelze, Germany, 99%) were mixed according to the stoichiometric formula and milled with zirconia balls in ethanol for 10 h at 200 rpm, using a planetary mill (Fritch Pulverissete 6, Idar-Oberstein, Germany). The resulting slurry was dried at 70 °C. Afterwards, the powder was calcined in alumina crucibles at 800 °C for 1 and 4 h.

For the solid state synthesis, two sets of sintered ceramics were obtained at low temperatures (970, 990, and 1000 °C for 5 h after synthesis at 800 °C for 4 h) and high temperatures (1020, 1050, and 1080 °C for 1 h after synthesis at 800 °C for 1 h).

In the Pechini method, the titanium solution was prepared by dissolving titanium isopropoxide (Sigma-Aldrich 97%) in absolute ethyl alcohol (J. T. Baker 99.9%) and added citric acid (Sigma-Aldrich 99.5%). In parallel, Ba(NO_3_)_2_ (Sigma-Aldrich 99%) and Na(NO_3_)_2_·4H_2_O (99.8%) were mixed using deionized water. In another container, Bi(NO_3_)_3_·5H_2_O and Ca(NO_3_)_2_·4H_2_O were mixed with ethylene glycol. Both preparations were added to the first solution. Finally, ethylene glycol (Sigma-Aldrich 99%) in a 4:1 M ratio with a citric acid was incorporated in the above solution. The solution was heated with constant stirring at 70 °C until it became transparent. Then, the solution was heated up to 120 °C, the point at which it became a dark-brown glassy resin. Charring the resin at 300 °C for 3 h resulted in a black solid mass that was lightly ground into a powder, using agate mortar. The powders obtained were heat treated at 600 and 800 °C in air for 4 h in alumina crucibles.

For the Pechini synthesis route, two other sets of sintered ceramics were obtained at low (1050, 1080 °C, and 1000 °C for 2.5 h after calcination at 600 °C for 4 h) and high temperatures (1100, 1120, 1150 °C for 1 h after synthesis at 800 °C—1 h for 4 h). Sintering was carried out in an electrical furnace.

For both synthesis methods, the powder was wet milled for 10 h at 200 rpm in a planetary mill and, after that, shaped into pellets of 12.7 mm in diameter by manual uniaxial pressing and then sintered.

Pellets aiming at determining dielectric and piezoelectric properties were polished at both sides and electrodes painted with silver paste, which was thermally treated at 500 °C for 30 min.

### 3.2. Characterization

The powders and sintered samples were characterized by X-ray diffraction (XRD) at room temperature using a Bruker D8 Advance diffractometer (Bruker Co., Billerica, MA, USA; Cu K_α1_ radiation, λ = 1.5406 Å) and goniometer with a Lynx eye (Bruker Co., Billerica, MA, USA) detector. The data were collected from 2 θ = 20° to 90° with 40 kV and 40 mA in the X-ray generator, step = 0.02° 2 θ and 38.4 s integration time to carry out a Rietveld analysis by using the TOPAS software version 4.1 [43].

Bulk densities of sintered ceramics were measured by the Archimedes method in water. Scanning Electron Microscopy (SEM) images were obtained by a JEOL 7600f (Peabody, MA, USA).

Samples for transmission electron microscopy (TEM) were prepared from powders suspended and ultrasonically dispersed in butanol. One drop of the suspension was placed on a copper grid coated by a holey carbon film. Selected-area electron diffraction and high-resolution TEM (HRTEM) experiments were performed using a JEOL 3000F (Tokio, Japan) microscope with a resolution limit of ≈1.1 Å. HRTEM images were recorded with an objective aperture of 70 μm centered on a sample spot within the diffraction pattern area. Fast Fourier transforms (FFTs) of the HRTEM images were carried out to reveal the periodic image contents using the Digital Micrograph package [44].

Relative dielectric permittivity, ε^T^_33_, and losses, tan δ, vs. temperature curves on heating and cooling were carried out in situ, i.e., with the sample inside the furnace, from complex impedance measurements at various frequencies in the range between 1 kHz and 1 MHz with a HP4194A analyzer. Disks were heated in an electrical furnace at a rate of 2 °C/min.

For piezoelectric characterization, the samples were thickness poled in a silicon oil bath under 4 KV/mm dc electric field at 50 °C for 30 min. All measurements were performed 24 h after the poling process. The piezoelectric parameter d_33_ was measured with a d_33_ piezometer system (PM300-PIEZOTEST, Singapore, Republic of Singapore). The d_31_ piezoelectric coefficient, as well as the electrochemical coupling factor k_P_, were calculated using the resonance method by an automatic iterative analysis [45] of the complex impedance spectra measured in an Agilent 4294A Precision Impedance Analyzer at the radial mode of thin disks, thickness poled.

## 4. Conclusions

Ceramics of the solid solution system (1 − *x*)Bi_0.50_Na_0.50_TiO_3_–*x*Ba_1__−_ *_y_*Ca_y_TiO_3_ (or BNBC100*y*T100*x*) with *x* = 0.06 and *y* = 0.10 (BNBC10T6) were obtained by two different synthesis routes: solid state and Pechini, succeeding in using reduced temperatures, both in synthesis (<800 °C) and sintering (<1150 °C). Dense ceramics (94–96% densification) were obtained by both routes, while keeping appropriated piezoelectric performance that did not correspond to the expected values for a Morphotropic Phase Boundary with coexistence of two ferroelectric phases, as observed at the tetragonal edge of the MPB. Ceramics from solid state show higher dielectric permittivity and d_33_ coefficients and lower planar electromechanical quality factors than those obtained by the Pechini route. Solid state ceramics show cubic grain morphology and Pechini ones show semispherical lower size grains.

Solid state synthesis produces ceramics that, as expected in comparison with the BNT-BT system, have a globally non-polar cubic phase, which consists of zones with local ferroelectric-rhombohedral-R3c and antiferroelectric-tetragonal-P4bm distortions. These ceramics undergo a field-induced phase transition to a ferroelectric rhombohedral structure (a = 3.9006 Å and α = 89.76°). Similarly to BNBT100*x* system for x ≥ 0.06, the dielectric permittivity curves as a function of temperature of non-poled solid state ceramics show a relaxor behavior from room temperature to the maximum of dielectric permittivity. Poled ceramics show a phase transition from the field-induced ferroelectric to the relaxor phase at ~100 °C.

In contrast, as revealed by XRD and TEM, the Pechini synthesis route produces BNBC10T6 ceramics with a spontaneous ferroelectric rhombohedral structure, similar to the BNBT100*x* system for x < 0.06. This has higher distortion (a = 3.8928 Å and α = 89.63°) than BNT or BNBT6. The dielectric permittivity curves as a function of temperature of non-poled Pechini ceramics show a phase transition from the spontaneous ferroelectric to an ergodic relaxor phase at 177 °C, well before the maximum of the dielectric permittivity takes place at ~250 °C. This phase transition shows thermal hysteresis and it is characteristic of the rhombohedral side (BNT-rich) of the MPB of BNT-based systems.

The structural and functional characteristics of these two sets of BNBC10T6 ceramics are dependent on the synthesis route, which reveals a, microstructure mediated, chemical shift of the MPB.

## Figures and Tables

**Table 1:** Preparation conditions, density, piezoelectric and dielectric parameters of the poled ceramics obtained by the solid state route.

Temp/Time	Temp/Time	Density	d_33_	d_31_	Kp	R.T.	R.T.
Synthesis (°C/h)	Sintering (°C/h)	(g/cm^3^)	(pC/N)	(pC/N)	(%)	ε^T^_33_	tan δ (%)
800/4	970/5	5.66	98	21	13	597	6
800/4	990/5	5.67	95	21	16	595	4
800/4	1000/5	5.79	20	5	4	591	40
800/1	1020/1	5.72	52	8	5	706	42
800/1	1050/1	5.65	68	18	13	642	5
800/1	1080/1	5.68	82	20	16	689	14

**Table 2:** Preparation conditions, density, piezoelectric and dielectric parameters of the poled ceramics obtained by the Pechini route.

Temp/Time	Temp/Time	Density	d_33_	d_31_	Kp	R.T.	R.T.
Synthesis (°C/h)	Sintering(°C/h)	(g/cm^3^)	(pC/N)	(pC/N)	(%)	ε^T^_33_	tan δ (%)
600/4	1050/2.5	5.64	81	19	20	319	2
600/4	1080/2.5	5.67	83	19	20	357	2
600/4	1100/2.5	5.75	74	19	20	359	2
800/4	1100/1	5.44	59	14	14	379	5
800/4	1120/1	5.64	77	15	12	538	2
800/4	1150/1	5.64	61	15	15	330	5

**Table 3:** Crystal lattice parameters obtained by the Rietveld analysis, size, and content of crystallites and quality factor (rwp) of the refinement.

Sample	Solid State		Pechini	
Phase Symmetry	R3c	Pm-3m	R3c	Pm-3m
As-sintered				
a (Å)	-	3.8964	3.8928	3.9229
α (°)	-	-	89.63	-
Crystal size (nm)	-	128	158	7
Content (%)	-	100	48.9	51.1
rwp	-	21.8	16.0	
Poled				
a (Å)	3.9006	-	3.8930	3.9221
α (°)	89.76	-	89.61	-
Crystal size (nm)	169	-	185	6
Content (%)	100	-	49.6	50.4
rwp	16.0	-	17.3	
Poled and powdered				
a (Å)	3.8962	-	3.8940	4.07
α (°)	89.76	-	89.62	
Crystal size (nm)	382	-	190	6
Content (%)	100	-	85.5	14.5
rwp	15.2	-	11.3	
